# Ferroptosis: Iron-mediated cell death linked to disease pathogenesis

**DOI:** 10.7555/JBR.37.20230224

**Published:** 2024-05-29

**Authors:** Xiangyu Zhang, Yingchao Hu, Bingwei Wang, Shuo Yang

**Affiliations:** 1 Department of Immunology, State Key Laboratory of Reproductive Medicine, Jiangsu Key Lab of Cancer Biomarkers, Prevention and Treatment, Collaborative Innovation Center for Personalized Cancer Medicine, Gusu School, the Affiliated Wuxi People's Hospital of Nanjing Medical University, Wuxi People's Hospital, Wuxi Medical Center, Nanjing Medical University, Nanjing, Jiangsu 211166, China; 2 Department of Pharmacology, Nanjing University of Chinese Medicine, Nanjing, Jiangsu 210023, China

**Keywords:** ferroptosis, lipid peroxidation, molecular regulatory mechanisms, ferroptosis-related diseases, ferroptosis inducers, ferroptosis inhibitors

## Abstract

Ferroptosis is a pattern of iron-mediated regulatory cell death characterized by oxidative damage. The molecular regulatory mechanisms are related to iron metabolism, lipid peroxidation, and glutathione metabolism. Additionally, some immunological signaling pathways, such as the cyclic GMP-AMP synthase-stimulator of the interferon gene axis, the Janus kinase-signal transducer and activator of transcription 1 axis, and the transforming growth factor beta 1-Smad3 axis, may also participate in the regulation of ferroptosis. Studies have shown that ferroptosis is significantly associated with many diseases such as cancer, neurodegenerative diseases, inflammatory diseases, and autoimmune diseases. Considering the pivotal role of ferroptosis-regulating signaling in the pathogenesis of diverse diseases, the development of ferroptosis inducers or inhibitors may have significant clinical potential for the treatment of aforementioned conditions.

## Introduction

In the past decade, ferroptosis has emerged as a pivotal mode of the programmed cell death, igniting some significant scientific interest. This cellular fate is morphologically, biochemically, and genetically distinct from its counterparts (*i.e.*, apoptosis, necrosis, and autophagy) and was first introduced in 2012^[1]^, representing a major advance in our understanding of cell death mechanisms. Ferroptosis is uniquely identified by the peroxidation of lipids on the cell membrane, a process associated with the iron-dependent reactions^[1]^. The peroxidation of membrane lipids is a defining feature of ferroptosis, distinguishing it from other forms of cell death and emphasizing the essential role played by iron in this process^[1–2]^.

Since its discovery, ferroptosis has attracted considerable scientific interest, with numerous extensive studies highlighting its critical role in a broad spectrum of pathological processes. These investigations have established the links between ferroptosis and various diseases, such as cancer, neurodegenerative disorders, and acute kidney injury, among others^[3–5]^. The distinctive features of ferroptosis, along with its potential association with a range of pathological conditions, have positioned it as a promising target for therapeutic intervention.

The intricate mechanisms underlying ferroptosis have become a pivotal focus of investigation. Investigators have delved into molecular mechanisms of ferroptosis, elucidating key regulatory pathways and identifying potential therapeutic targets. The glutathione peroxidase 4 (GPX4) enzyme, for instance, has emerged as a critical regulator of ferroptosis, catalyzing the reduction of lipid peroxides and preventing cell death^[6]^. Additionally, the role of iron metabolism in ferroptosis has come under scrutiny, with studies implicating iron overload as a potential trigger for this form of cell death^[2,7]^.

The present review aims to contribute to this burgeoning field by providing a comprehensive overview of the regulatory frameworks, molecular mechanisms, and therapeutic implications associated with ferroptosis. Because we forge ahead in our study of ferroptosis-associated maladies, we hope to pave the way for new theoretical groundwork and exploratory avenues that will ultimately improve patient outcomes.

## The overview of ferroptosis

### The discovery of ferroptosis

In 2003, Dolma *et al*^[8]^ discovered and named the compound erastin during the synthesis and subsequent performance of the screening experiments for anti-tumor drugs. Erastin induced cell death in tumor cells, but the type of cell death was distinct from apoptosis, as there was no nuclear shrinkage or activation of caspase 3 in this process^[8]^. In 2008, Yang *et al*^[2]^ discovered that the use of antioxidant vitamin E and iron chelator deferiprone (DFP) inhibited erastin-induced cell death, and the key regulators of iron metabolism, such as transferrin (TF) and TF receptor (TFR), were also involved. The involvement of reactive oxygen species (ROS) and the Fenton reaction in the process of cell death mentioned above was also revealed^[2]^. In 2012, Dixon *et al*^[1]^ summarized and elucidated the process of erastin-induced cell death, and found that during this process, the uptake of cysteine by the cell surface cysteine/glutamate antiporter (system X_c_^–^) was reduced, the glutathione (GSH)/GPX4 axis was abnormal, and intense lipid peroxidation reaction occurred in the polyunsaturated fatty acids (PUFAs) on the cell membrane. Therefore, the iron-dependent cell death pattern caused by oxidative damage was named ferroptosis^[1]^. It was found later that the peroxidation reaction mechanism of PUFAs mediated by lipoxygenases (LOXs) also participated in ferroptosis^[9]^. In summary, ferroptosis is a regulated cell death that is associated with the redox state of cells. Iron metabolism, amino acid metabolism related to the GSH synthesis, the GSH-GPX4 antioxidant axis, the Fenton reaction, and LOXs-mediated peroxidation of PUFAs are all involved in the processes of ferroptosis.

### Characteristics of ferroptosis and its differences from other types of regulated cell death

Ferroptosis is a programmed cell death that differs from other types of cell death, such as apoptosis, necrosis, autophagy, and pyroptosis. Cells undergoing ferroptosis exhibit distinct morphological and biochemical characteristics (***Table 1***). The morphological changes mainly occur in mitochondria, characterized by the accumulation of homogeneous heteromorphic small mitochondria with increased dense material, increased membrane density, ruptured outer membrane, reduced volume, and diminished or absent ridge structures^[1,10]^. In addition to these mitochondrial changes, morphological alterations also occur in cell membranes during ferroptosis.

**Table 1:** Comparison of characteristics of different types of regulatory cell death

Types	Morphological features	Biochemical features
Necroptosis	Cell swelling, membrane structure broken^[162]^, cell fragments formed, organelles expanded and deformed, and moderate chromatin condensation	DAMPs release^[163]^, intracellular peroxide content increases, and NADPH content decreases
Apoptosis	Cell shrinkage, cell membrane blister, complete membrane structure, nuclear pyknosis, nucleolus fragmentation, chromatin agglutination, and apoptosis body formation	Without DAMPs release, the phosphatidylserine in the inner cell membrane turned over to the outer membrane, and phosphatidylserine oxidation^[164]^, non-random degradation of genomic DNA^[165]^, and mitochondrial membrane potential decreased
Pyroptosis	Cell swelling^[166]^, cell membrane blister, and holes formation in the inner side of the cell membrane^[167]^	Formation of inflammasome, DAMPs, and pro-inflammatory cytokines IL-1β and IL-18 release
Ferroptosis	Increased cell membrane density, pore formation on cell membranes, the nucleus remains intact, smaller mito-chondria, decreased mitochondria crista, and elevated mitochondrial membrane densities^[1]^	Mitochondrial membrane potential decreased, ROS level increased significantly, intracellular Fe^2+^ accumulated, GSH, GPX4, and cystine levels decreased, and lipid peroxidation products accumulated^[14]^
Autophagy	Normal cell volume, intact membrane structure, no chromatin condensation, autophagic vacuolization^[168]^, mitochondria and endoplasmic reticulum swollen, and Golgi apparatus enlarged	Elevated lysosomal activity and increased levels of autophagy-related proteins, such as ATG5, ATG7, and p62
Abbreviations: ATG5, autophagy-related protein 5; ATG7, autophagy-related protein 7; DAMPs, damage-associated molecular patterns; DNA, deoxyribonucleic acid; GPX4, glutathione peroxidase 4; GSH, glutathione; IL-1β, interleukin-1beta; IL-18, interleukin-18; NADPH, nicotinamide adenine dinucleotide phosphate; p62 (SQSTM1), sequestosome 1; ROS, reactive oxygen species.		

Unlike apoptosis, where the cell membrane blebs and retains its structural integrity^[11]^, ferroptotic cells exhibit increased membrane density and membrane rupture. Furthermore, unlike necrosis and pyroptosis, ferroptosis does not result in cell swelling or enlargement^[12]^. In the process of ferroptosis, the nucleus remains intact and normal in size, without chromatin agglutination, which is different from the phenomenon of nucleus concentration, nuclear membrane and nucleolus fragmentation, and chromatin aggregation in apoptosis^[11–12]^.

During ferroptosis, cells undergo several distinct alterations in their biochemical indicators. Notably, they experience a significant depletion of adenosine triphosphate (ATP), accompanied by elevated levels of Fe^2+^ and ROS^[6,13]^. Concurrently, the levels of GSH and cysteine decrease, while GPX4 expression is significantly downregulated^[6]^. Furthermore, lipid peroxidation within bio-membranes is amplified, primarily manifesting as the peroxidation of phosphatidylethanolamine within the membrane itself^[14]^. Moreover, hyperoxidized peroxiredoxin 3, the first specific biochemical marker of ferroptosis, has been identified^[15]^. In the process of ferroptosis, mitochondrial lipid peroxides trigger peroxiredoxin 3 peroxidation and convert cysteine thiols to sulfinic or sulfonic acids^[15]^. Once peroxiredoxin 3 is hyperoxidized, it translocates from the mitochondria to the plasma membrane, where it inhibits cystine uptake, thereby causing ferroptosis^[15]^. Ferroptosis unveils a complex interplay of biochemical alterations, highlighting the significance of oxidative stress in cell death mechanisms.

### Ferroptosis-related signals

The triggering of ferroptosis is related to lipid metabolism and iron metabolism, and the signaling pathways mainly involved in ferroptosis include the synthesis and oxidation of PUFAs, the processing and transport of extracellular iron, and the storage and release of intracellular iron^[6]^. Key regulators in the above-mentioned metabolic pathways include acyl-CoA synthetase long-chain family member 4 (ACSL4), lysophosphatidylcholine acyltransferase 3 (LPCAT3), LOXs, TFR, TF, ferritin, and nuclear receptor coactivator 4 (NCOA4).

The mechanism of ferroptosis resistance mainly involves a variety of intracellular antioxidant signaling pathways, including the cysteine-GSH-GPX4 pathway^[16]^, NADPH-ferroptosis suppressor protein 1 (FSP1)-coenzyme Q_10_ (CoQ_10_) pathway^[17]^, dihydroorotate dehydrogenase (DHODH)-CoQ_10_ antioxidant signaling pathway^[18]^, guanosine triphosphate (GTP) cyclohydrolase 1 (GCH1)-tetrahydrobiopterin (BH4)-dihydrofolate reductase (DHFR) antioxidant signaling pathway^[19]^, and p62-Kelch-like ECH-associated protein 1 (KEAP1)-nuclear factor erythroid 2-related factor 2 (NRF2) pathway^[20]^. Key regulatory factors in the above antioxidant signaling pathways include solute carrier family 7 member 11 (SLC7A11), solute carrier family 3 member 2 (SLC3A2), GPX4, FSP1, DHODH, GCH1, DHFR, and NRF2.

In addition to metabolic and antioxidant dimensions, ferroptosis regulation is also influenced by immune-related signaling cascades. Specifically, the cyclic GMP-AMP synthase (cGAS)-stimulator of interferon genes (STING)^[21]^, Janus kinase (JAK)-signal transducer and activator of transcription 1 (STAT1)^[22–23]^, and transforming growth factor-beta 1 (TGF-β1)-SMAD3 signaling pathways have been implicated in the delicate orchestration of ferroptosis^[24]^. This interplay underscores the complex interplay among metabolism, redox biology, and immunity in shaping the fate of cells undergoing ferroptosis.

### Pathophysiological significance of ferroptosis

Ferroptosis is involved in various pathological processes, particularly in tumor development^[25–29]^, as well as neurodegenerative disorders, such as Alzheimer's disease (AD)^[30–31]^ and Parkinson's disease (PD)^[32]^. It also plays a pivotal role in immune-inflammatory diseases and autoimmune diseases^[33–36]^. The onset and progression of these diseases are linked with ferroptosis. Later in the present review, we will provide a detailed examination of the specific roles and implications of ferroptosis in these various pathological processes.

## Regulatory signaling mechanisms of ferroptosis

**Figure 1:**
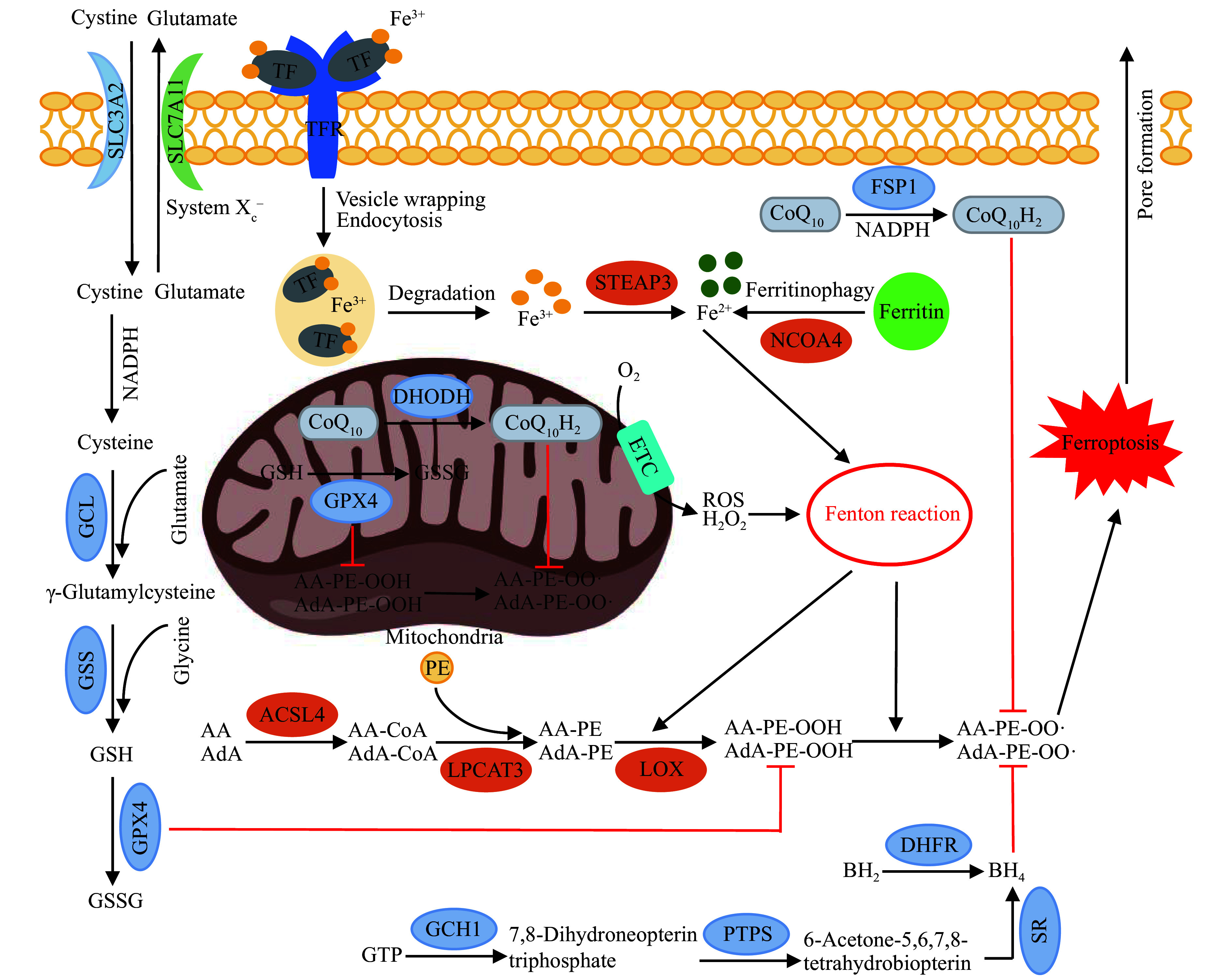
The mechanisms underlying the formation of ferroptosis.

The ferroptosis process mainly involves the following steps: disruption of the iron metabolism leading to the Fenton reaction induction, imbalance of redox homeostasis, and triggering of lipid peroxidation reactions^[6]^. When the disorder of iron metabolism induces the Fenton reaction, and the cellular antioxidant defense mechanism is not sufficient to clear the large amount of strong oxidative free radicals produced by the Fenton reaction, it will cause severe lipid peroxidation of membrane lipid components, thereby triggering ferroptosis^[37–38]^. Concerning the three processes mentioned earlier, we have summarized the various mechanisms involved in the regulation of ferroptosis signaling in ***Fig. 1***.

### Iron metabolism and Fenton reaction

The Fenton reaction is an inorganic chemical reaction, in which H_2_O_2_ reacts with a large amount of Fe^2+^ to generate highly oxidizing hydroxyl radicals (·OH). The hydroxyl radicals then trigger lipid peroxidation in the cell membrane, ultimately leading to cell membrane damage and cell death. The ROS required for the Fenton reaction are primarily generated in mitochondria^[38]^. Additionally, NADPH cytochrome P450 reductase and NADH cytochrome b5 reductase, localized on the endoplasmic reticulum membrane, facilitate electron transfer from NAD(P)H to O_2_, resulting in the production of H_2_O_2_^[39]^. Fe^2+^ mainly comes from the direct degradation of intracellular ferritin, as well as the transportation and processing of extracellular iron by TF.

The main form of extracellular iron is Fe^3+^. In the process of transporting and processing extracellular iron, TF carries two molecules of Fe^3+^ and binds to TFR on the surface of the cell membrane, then is encapsulated by vesicles and endocytosed into the cytoplasm, promoting the release of Fe^3+^ in a low pH environment of the vesicles^[40– 41]^. Free Fe^3+^ is reduced to Fe^2+^ by the six-segment transmembrane epithelial antigen of prostate 3, and Fe^2+^ plays a variety of biological functions in the cytoplasm or is stored in ferritin that is composed of ferritin light chain and ferritin heavy chain 1^[40–41]^.

The degradation of intracellular ferritin requires the participation of NCOA4, which is indispensable for the transport of ferritin to lysosomes^[42]^. Under the regulation of NCOA4, ferritin is selectively degraded through autophagic lysosomes, releasing a large amount of Fe^2+^ to regulate intracellular iron metabolism, a process called ferritinophagy^[42–43]^. Knockout of proteins related to the autophagy process, such as autophagy-related 5 (ATG5), ATG7, and NCOA4, inhibits ferritinophagy, thereby inhibiting ferroptosis, while overexpression of NCOA4, on the other hand, promotes ferritinophagy and induces cell ferroptosis^[44]^. In general, an elevation in intracellular iron concentrations, either by enhancing intracellular ferritin breakdown or boosting extracellular iron absorption, results in an overload of Fe^2+^. This overload facilitates the initiation of the Fenton reaction, consequently driving ferroptosis progression.

### Antioxidant system

Under physiological conditions, various antioxidant signaling pathways maintain cellular redox homeostasis and prevent cellular damage caused by oxidative stress.

#### GSH metabolism and Cys-GSH-GPX4 axis

GSH is the most abundant antioxidant in the body, while GPX4 is the only GSH peroxidase responsible for the reduction of lipid peroxides in cells^[45]^. Both GSH and GPX4 play key roles in cellular antioxidant defense. GPX4 has been identified as a crucial factor in ferroptosis^[16]^, which converts the reduced GSH into glutathione disulfide (GSSG), while simultaneously converting the peroxidized bond of lipid hydroperoxides into a hydroxyl group, generating the corresponding alcohol and thus eliminating peroxidative activity^[46]^. This process helps prevent lipid peroxidation reactions caused by an excessive oxidative stress, thereby protecting the cell membrane. In addition to the role of antioxidant and free radical scavenger, GSH also acts as a cofactor of GPX4 and works synergistically with GPX4 to counteract lipid peroxidation reactions^[47]^. Therefore, an inadequate GSH synthesis directly or indirectly impairs the antioxidant defense by inhibiting the GPX4 activity^[48]^. The dynamic antioxidant duo, with the abundant presence of GSH complementing GPX4's unique peroxidase role in cellular lipid peroxide reduction and synergistic defense against ferroptosis, emphasizes the crucial interplay between antioxidants for maintaining cellular homeostasis.

The efficiency of GSH synthesis is limited by the concentration of cysteine, with glutamate-cysteine ligase and GSH synthetase serving as the rate-limiting enzymes during the process^[49–50]^. Cysteine required for the GSH synthesis is mainly transported to the cytoplasm through the cell surface system X_c_^–^, a heterodimeric cysteine/glutamate antiporter that is widely distributed in the phospholipid bilayer^[51]^. The transport system is mainly composed of SLC7A11 and SLC3A2, which are connected by a disulfide bond. This system transports extracellular cystine into the cytoplasm, while simultaneously transports intracellular glutamate out of the cytoplasm in the same proportion^[51]^. Subsequently, cystine is reduced to cysteine under the action of NADPH, and then, with the catalysis of glutamate-cysteine ligase and GSH synthetase, it is further converted into GSH^[52]^.

As the major cellular antioxidant defense mechanism, the Cys-GSH-GPX4 axis plays an important role in maintaining redox homeostasis in cytoplasm and mitochondria. Blocking the function of system X_c_^–^ or inhibiting the activity and expression of glutamate-cysteine ligase and GSH synthetase effectively promotes ferroptosis^[46,53]^. In addition to SLC7A11 and SLC3A2, other SLC family members, such as SLC47A1^[54]^, are also involved in the regulation of ferroptosis; however, their involvement differs from that of system X_c_^–^. Mechanistically, SLC47A1 exerts ferroptosis resistance mainly by affecting lipid remodeling during ferroptosis^[54]^. SLC47A1 acts as an endogenous repressor of ferroptosis, presumably by affecting the concentrations of PUFAs as well as their metabolism^[54]^.

Cysteine scarcity governs GSH synthesis efficacy, while the SLC family orchestrates ferroptosis regulation, emphasizing the intricate Cys-GSH-GPX4 interplay for redox homeostasis and the delicate balance between antioxidant defense and lipid metabolism in preserving cellular integrity.

#### FSP1-cytoplasm CoQ_10_-NAD(P)H axis

The reduced form of CoQ_10_H_2_, known as ubiquinol, functions as a lipophilic electron carrier in the inner mitochondrial membrane. CoQ_10_, when in its reduced form as ubiquinol, captures oxygen free radicals that participate in lipid peroxidation reactions. Through oxidation, ubiquinol is converted back to its oxidized form, CoQ_10_, thereby completing the cycle of antioxidant activity^[17]^. FSP1 is a NAD(P)H-dependent oxidoreduction enzyme located in the plasma membrane. As a key component of the non-mitochondrial CoQ antioxidant system, FSP1 facilitates the reduction of CoQ_10_ to CoQ_10_H_2_ by NAD(P)H, thereby promoting the regeneration of CoQ_10_H_2_ in the cytoplasm through the FSP1-cytosolic CoQ_10_-NAD(P)H axis^[17]^. As an independent antioxidant system, the FSP1-CoQ_10_-NAD(P)H pathway works in parallel with the cytosolic GPX4-GSH antioxidant system to resist ferroptosis^[17]^, and this signal axis may partially meet the antioxidant demand of cells in the absence of GPX4.

#### DHODH-mitochondrial CoQ_10_ axis

DHODH mediates the mitochondrial anti-ferroptosis defense system, which acts in parallel with the mitochondrial GSH-GPX4 antioxidant system to counteract lipid peroxidation reactions in the mitochondria. DHODH, localized on the outer surface of the inner mitochondrial membrane^[55]^, catalyzes the reduction of CoQ_10_ in the mitochondria to CoQ_10_H_2_^[18]^, forming a DHODH-mitochondrial CoQ_10_ axis. Under normal conditions, the cytoplasmic and mitochondrial antioxidant defenses are mainly executed by the GPX4-GSH axis. Nevertheless, when GPX4 is inactivated or the GSH content is insufficient, the DHODH content significantly increases, promoting the generation of CoQ_10_H_2_ in the mitochondria and enhancing the capture of oxygen free radicals^[18]^. However, there is still controversy regarding the inhibitory effect of DHODH on ferroptosis. One study has shown that the inhibitory effect of DHODH on ferroptosis is associated with FSP1^[56]^. The ferroptosis-sensitizing effect of brequinar (and several other DHODH inhibitors) is mediated *via* the inhibition of FSP1 at high concentrations but not *via* DHODH^[56]^. DHODH synergizes with the GSH-GPX4 antioxidant axis to counteract mitochondrial lipid peroxidation, strengthening its role, when GPX4 is compromised or GSH content is depleted. However, the inhibitory effect of DHODH on ferroptosis remains contentious and is linked to FSP1 involvement, thus highlighting the delicate equilibrium between mitochondrial defenses and ferroptosis regulation.

#### GCH1-BH4-DHFR axis

BH4 scavenges lipid peroxide radicals while being oxidized to dihydrobiopterin (BH2)^[19]^. The biosynthesis of BH4 occurs through two pathways: salvage synthesis and *de novo* synthesis. The salvage synthesis pathway refers to the process where BH2 is reduced back to BH4 by DHFR using NADPH as the source of reducing power. The *de novo* synthesis process starts with GTP and sequentially involves the catalytic actions of GCH1, 6-pyruvoyltetrahydropterin synthase (PTPS), and sepiapterin reductase to produce BH4^[57]^. In the *de novo* synthesis pathway, GCH1 acts as a rate-limiting enzyme that regulates the production of BH4. Increasing the expression or activity of GCH1 enhances the synthesis of BH4, and accelerates the clearance of lipid peroxidation free radicals. Additionally, there have been reports indicating that BH4 accelerates the conversion of phenylalanine to tyrosine, promotes the generation of 4-hydroxybenzoic acid ester, which is a precursor of CoQ_10_ biosynthesis, and thus intervenes in the regulation pathway of CoQ_10_-related anti-lipid peroxidation^[58]^.

#### p62-KEAP1-NRF2 axis

NRF2 is involved in the resistance to ferroptosis through its ability to inhibit iron uptake, limit ROS production, and upregulate SLC7A11 expression^[20]^. Under basal conditions, NRF2 is maintained at low levels through KEAP1-mediated NRF2 ubiquitination and proteasomal degradation^[59]^. Under oxidative stress, the autophagy receptor p62 is highly expressed and interacts with the NRF2 binding site on KEAP1, competitively inhibiting the KEAP1-NR2 binding, and releasing the negative regulation of NRF2 by KEAP1^[59]^. The accumulation of NRF2 in the nucleus and its activation leads to the promotion of gene expression, including antioxidant response elements, such as heme oxygenase-1 (*HMOX-1*), ferritin heavy chain (*FTH1*), and NADPH quinone oxidoreductase 1 (*NQO1*)^[59–60]^. The expression of these genes plays a role in inhibiting ferroptosis^[59–60]^. However, there are also reports indicating that overexpression of some NRF2 target genes may induce ferroptosis. For example, HMOX-1 functions as an antioxidant enzyme that degrades heme groups and mitigates their pro-oxidant effects. However, research on neuroblastoma cells has found that overexpression of HMOX-1 paradoxically results in excessive degradation of heme, leading to the release of a large amount of Fe^2+^ ions and cellular iron overload, and subsequently inducing ferroptosis^[61]^.

### Lipid metabolism and lipid peroxidation

When the above-mentioned antioxidant system is insufficient to counteract oxidative stress caused by the Fenton reaction, it triggers intense lipid peroxidation reactions, leading to ferroptosis. Lipid peroxidation refers to the oxidative degradation of lipid components in the cell organelle membrane and the cell membrane, which occurs because of free radical removal of hydrogen or the loss of hydrogen atoms under the action of peroxidase, leading to oxidation, breakage, and shortening of the carbon chain of lipids, generating toxic substances like lipid free radicals, lipid hydroperoxides, and reactive aldehydes (*e.g*., malondialdehyde and 4-hydroxynonenal), which induce cell toxicity and ultimately cause cell damage^[62]^. Lipid peroxidation and the accumulation of its oxidation products are the hallmarks of ferroptosis^[9]^. This reaction, as a core component of ferroptosis, may cause oxidative degradation of cell membrane components, playing a crucial role in the integrity of cell membrane and the survival or death of the cell.

#### ACSL4-LPCAT3-AA/AdA-PEs axis

The primary substrates for lipid peroxidation are PUFAs and phosphatidylethanolamines (PEs), which are abundant in PUFAs within biological membranes. These PUFAs possess bis-allylic hydrogens that exhibit relatively low bond dissociation energies in comparison to other lipid molecules, rendering them susceptible to peroxidation reactions^[9]^. The amount of PUFAs in the membrane lipids determines the degree of lipid peroxidation and the sensitivity of cells to ferroptosis. Expression of the elongated very-long-chain fatty acid protein 5 and fatty acid desaturase has been found to enhance the synthesis of more elongated and desaturated PUFA species that promote ferroptosis sensitivity^[63–64]^. As a rate-limiting enzyme in the β-oxidation process, 2,4-dienoyl-CoA reductase 1 (DECR1) is involved in the catabolism of PUFAs in mitochondria, and the loss of DECR1 function may lead to the accumulation of intracellular PUFAs and increase ferroptosis sensitivity^[65]^. However, exogenous monounsaturated fatty acids (MUFAs) inhibit the entry of PUFAs into membrane lipids and reduce the generation of lipid peroxidation free radicals, thus playing a protective role against ferroptosis, and this process entails the activation of MUFA by acyl-CoA synthetase long-chain family member 3 (ACSL3)^[66]^. Membrane-bound O-acyltransferase 2 acts as a GPX4/FSP1-independent inhibitor of ferroptosis, and it selectively enhances the generation of PE-containing MUFAs, while reducing the generation of PE-containing PUFAs^[67]^. Through this PE remodeling, it drives cells towards a state with high levels of PE-MUFA but low levels of PE-PUFA, leading to the resistance against ferroptosis. In summary, the synthesis of MUFAs mediated by ACSL3 and the synthesis of PUFAs mediated by ACSL4 play different roles in ferroptosis, with the former hindering lipid peroxidation but the latter promoting lipid peroxidation and ultimately triggering ferroptosis.

The synthesis of PUFAs is primarily regulated by ACSL4 and LPCAT3. ACSL4 tends to bind to long-chain PUFAs and catalyzes the ATP-dependent esterification of arachidonic acid (AA) and adrenic acid (AdA) into AA-CoA and AdA-CoA, respectively^[68–69]^. Subsequently, AA-CoA and AdA-CoA, under the catalysis of LPCAT3, combine with PEs on the cell membrane to form the main substrate of lipid peroxidation, AA-PE and AdA-PE, respectively^[70]^. As key enzymes regulating the synthesis and remodeling of PUFAs in phospholipid membranes^[61]^, the lack or decreased activity of ACSL4 and LPCAT3 may hinder the synthesis of substrates in the lipid oxidation process, reducing the sensitivity of cells to ferroptosis. As an example of the ACSL4 enzyme activity regulation, the PKC family member PKCβⅡ participates in the regulation of lipid peroxidation by enhancing the ACSL4 activity^[71]^. PKCβⅡ directly interacts with ACSL4 and phosphorylates it at threonine 328, activating ACSL4 and enhancing the biosynthesis of PUFA-containing lipids, thereby promoting the generation of lipid peroxidation products^[71]^. Subsequently, the lipid peroxidation-PKCβⅡ-ACSL4 positive feedback loop amplifies lipid peroxidation and ultimately leads to ferroptosis^[71]^.

#### Lipid peroxidation

The initial step of membrane phospholipid peroxidation is the generation of lipid hydroperoxides (LOOH). LOOH is primarily produced through two pathways: a non-enzymatic pathway mediated by free radicals and an enzymatic pathway mediated by lipoxygenases (LOXs)^[72–73]^. The non-enzymatic pathway for LOOH generation requires the involvement of ROS, as roughly described as follows: hydroxyl radicals produced by the Fenton reaction combine with a hydrogen atom from PUFAs in the membrane phospholipids, leading to the formation of a lipid radical (L·), but the lipid radical is unstable and reacts further with an oxygen molecule to produce a lipid peroxide radical (LOO·)^[72]^, and then the LOO· takes a hydrogen atom from neighboring PUFAs in the lipids to form a LOOH and a lipid radical^[72]^. The newly formed lipid radical further reacts with an oxygen molecule to trigger a lipid radical chain reaction, leading to a cascade of lipid oxidation reactions and the generation of intermediate products, such as malondialdehyde and 4-hydroxynonenal^[9]^. These products are cytotoxic and cause abnormal covalent modifications of proteins in nucleic acids and cell membranes, resulting in the inactivation of proteins involved in normal physiological functions and promoting the occurrence of ferroptosis^[9]^.

The enzymatic pathway for LOOH synthesis is mediated by LOXs, which are a family of iron-containing non-heme dioxygenases^[74]^. Human LOX family consists of six subtypes (*i.e.*, 5-LOX, 12-LOX, 12B-LOX, 15-LOX, 15B-LOX, and E3-LOX), whereas mice have seven subtypes (*i.e.*, 5-LOX, 12-LOX, 12b-LOX, 15-LOX, 15b-LOX, e3-LOX, and 12e-LOX)^[74]^. These enzymes mainly catalyze the interaction between oxygen and molecules, such as AA and AdA to generate LOOH. It is known that LOX, especially the 15-LOX isoform, plays a critical role in regulating ferroptosis^[73]^. For example, the 15-LOX may bind to phosphatidyl ethanolamine-binding protein 1 (PEBP1) and undergo conformational regulation, specifically oxidizing AA/AdA-PE on the membrane to AA/AdA-PE-OOH, which in turn triggers ferroptosis^[75]^.

Apart from the fatty acid metabolic processes mentioned earlier and implicated in ferroptosis regulation, bile acid metabolism also connects to ferroptosis. For example, Tschuck *et al*^[76]^ have shown that the activation of the nuclear receptor Farnesoid X receptor, triggered by bile acids, leads to the upregulation of pivotal genes, namely *GPX4*, *FSP1*, and *ACSL3*, which curtail ferroptosis. In essence, the network of lipid metabolism plays a pivotal role in both the initiation and modulation of lipid peroxidation during ferroptosis^[76]^. A more profound comprehension of the interplay between the lipid metabolism network and ferroptosis may shed light on the mechanisms underlying ferroptosis and pave the way for the development of targeted therapeutic strategies.

### Immune-related signaling pathways are involved in the regulation of ferroptosis

**Figure 2:**
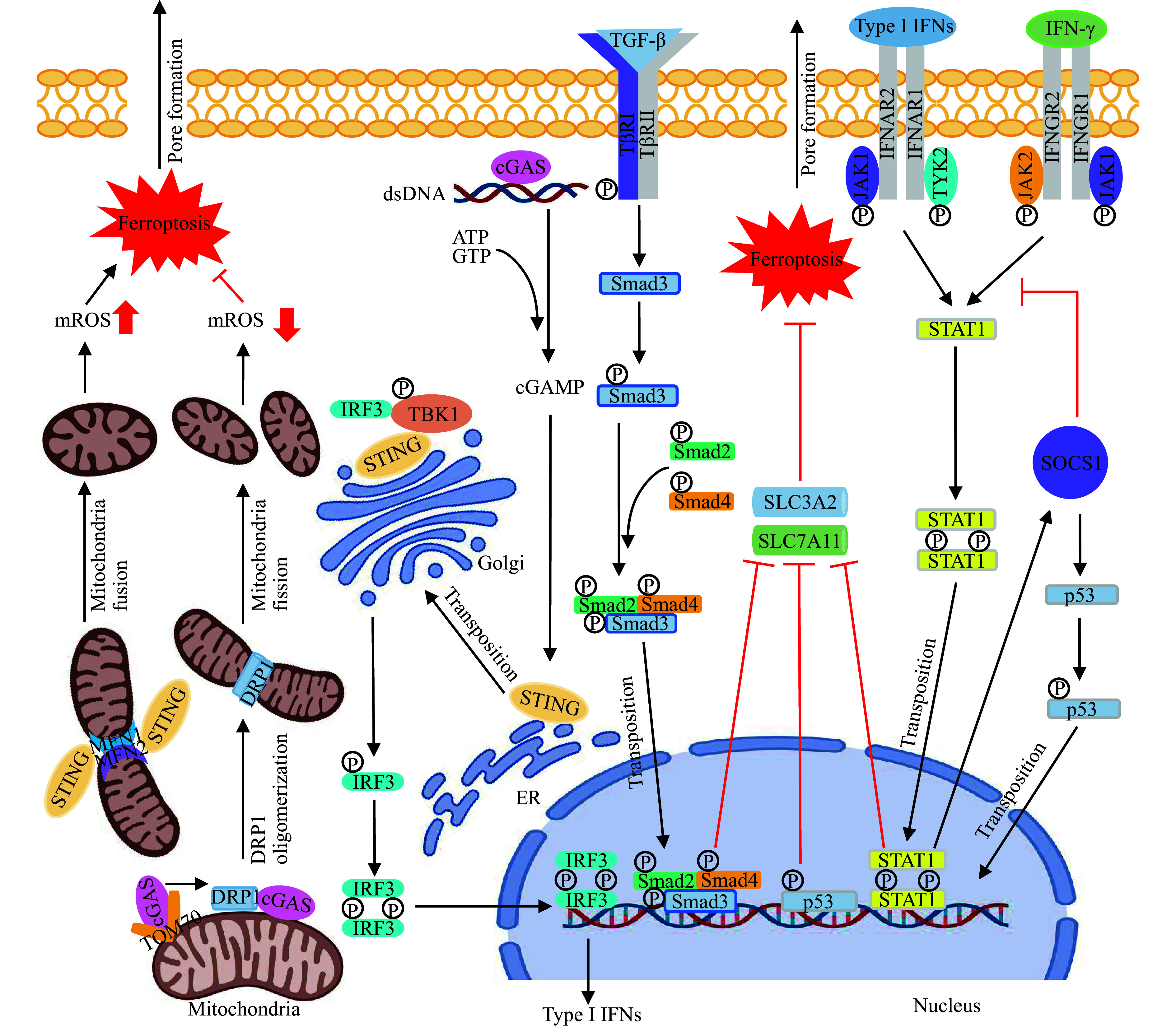
Immune signaling pathways implicated in the modulation of ferroptosis.

Besides various metabolic pathways involved in the regulation of ferroptosis, some immune-related signaling pathways also contribute to the regulation of ferroptosis sensitivity (***Fig. 2***).

#### STING-MFN1/2 axis

cGAS is a recently discovered innate immune sensor that recognizes DNA. It catalyzes the formation of 2′3′-cyclic GMP-AMP (cGAMP) and activates the signaling pathway relying on STING that mediates the transcriptional expression of IFN-I and IFN-stimulated genes (ISGs), thus participating in the antiviral innate immune response^[77–80]^. One study has demonstrated that ferroptosis influences the activation of the cGAS-STING signaling pathway, and the lipid oxidation-reduction homeostasis maintained by GPX4 is a necessary factor for the STING activation^[81]^. The lipid peroxidation metabolite 4-hydroxynonenal promotes carbonylation modification of cysteine 88 in STING, thereby inhibiting STING palmitoylation and the translocation from the endoplasmic reticulum to the Golgi apparatus, which hinders the activation of the cGAS-STING signaling pathway^[81]^. The main substrate of lipid peroxidation, PUFAs, are also associated with cGAS-STING signaling transduction. Through molecular docking analysis and *in vitro* experiments, Vila *et al*^[21]^ demonstrated that PUFAs specifically bond to the cGAMP-binding domain of STING, thereby blocking downstream TANK binding kinase 1 and interferon regulatory factor 3 phosphorylation and inhibiting STING-mediated inflammatory response. Additionally, STING may impede the formation of PUFAs by inhibiting the fatty acid desaturase 2-associated desaturation activity^[21]^, thereby hindering the occurrence of ferroptosis^[64]^. In summary, intracellular lipid peroxidation substrates and products may impede the cGAS-STING signaling pathway, while STING may hinder ferroptosis by disrupting the PUFA synthesis. This interplay of regulators is crucial for maintaining a balance between inflammation and ferroptosis, especially in pathological conditions.

In addition, STING may regulate mitochondrial dynamics by activating the STING-mitofusin 1 and 2 (MFN1/2) signaling, which in turn affects ferroptosis. In human pancreatic cancer cell lines, for example, studies showed that STING was translocated from the endoplasmic reticulum to mitochondria to form a STING-MFN1/2 complex by binding with MFN1/2 proteins on the outer mitochondrial membrane^[82–83]^. As key effectors of the mitochondrial outer membrane fusion, MFN1/2 may promote the accumulation of mitochondrial ROS (mtROS) by inducing the mitochondrial outer membrane fusion, ultimately leading to lipid peroxidation^[82–83]^. In summary, the activation of the STING-MFN1/2 pathway promotes ferroptosis.

#### cGAS-DRP1 axis

It has been shown that cGAS participates in regulating ferroptosis and promoting the development of hepatocellular carcinoma (HCC), independent of the classical cGAS-STING signaling pathway^[84]^. Qiu *et al*^[84]^ discovered that cGAS was relocalized to the outer mitochondrial membrane of HCC cells *via* the mitochondrial outer membrane protein TOM70. Subsequently, cGAS directly binds with dynamin-related protein 1 (DRP1), a protein involved in regulating mitochondrial membrane fission, and promotes DRP1 oligomerization^[84–85]^. This process induces the mitochondrial outer membrane fission and inhibits the accumulation of mtROS, thereby conferring resistance to ferroptosis and promoting tumor growth^[84–85]^.

#### JAK-STAT1 axis

CD8^+^ T cells in the tumor microenvironment release interferon-gamma (IFN-γ) that binds to the IFN-γ receptor on the surface of tumor cells, and then activates the JAK-STAT1 signaling pathway to increase the sensitivity of tumor cells to ferroptosis^[86–87]^. In the above-mentioned pathways, STAT1 is activated and translocated to the nucleus in the form of a dimer that acts as a transcriptional repressor, inhibiting SLC7A11 and SLC3A2 expression, and thus reducing the resistance of tumor cells to lipid peroxidation^[86–87]^.

In the JAK-STAT signaling pathway, the activated STATs upregulate the suppressor expression of cytokine signaling 1 (SOCS1) that binds to phosphorylated JAKs and cytokine receptors, thus specifically blocking the JAK activity and closing the JAK-STAT signaling pathway^[88]^. In addition to the above-described functions, some studies have reported that the SOCS1-p53 axis is also involved in the regulation of ferroptosis, because SOCS1 promotes p53 phosphorylation, which acts as a transcriptional repressor to inhibit SLC7A11 expression, thereby improving the sensitivity of tumor cells to ferroptosis^[89–90]^. Furthermore, the 12-LOX is also involved in the p53-dependent ferroptosis regulation mechanism^[91]^. In normal conditions, the 12-LOX maintains low activity by binding to SLC7A11, while p53 inhibits the expression of SLC7A11, resulting in the dissociation of 12-LOX from SLC7A11, and thus inducing lipid peroxidation and mediating ferroptosis under oxidative stress^[91]^.

The interplay between CD8^+^ T cells and ferroptosis pathways in tumor microenvironment underscores the potential of harnessing this mechanism for therapeutic benefit. Modulating lipid peroxidation sensitivity through the JAK-STAT1 pathway and the SOCS1-p53 axis may offer new avenues for innovative cancer treatments by emphasizing the significance of immune-mediated lipid peroxidation in tumor suppression.

#### TGF-β1-SMAD3 axis

TGF-β1 is known to be related to tumorigenesis. In early tumor stages, TGF-β1 may trigger tumor cell apoptosis and inhibit tumor cell proliferation; however, in advanced tumors, the high concentration of TGF-β1 in the tumor microenvironment promotes tumor progression by initiating immune escape, inducing epithelial-to-mesenchymal transition, and thus promoting tumor angiogenesis^[92–93]^. It has been shown that the inhibitory effect of TGF-β1 on early tumor progression may also be related to the mechanism of ferroptosis^[94]^, because TGF-β1 inhibits the expression of system X_c_^–^ by activating the downstream classical Smad-dependent signaling pathway, thereby enhancing lipid peroxidation in early HCC cells and promoting tumor clearance through the ferroptosis-related mechanisms^[94]^. In addition, bone morphogenetic protein-7, as a well-known antagonist of TGF-β, has been shown to impede the progression of diabetic nephropathy by inhibiting the canonical TGF-β pathway, attenuating ferroptosis, and helping regenerate the diabetic pancreas^[24]^.

When the antioxidant axis is imbalanced and fails to efficiently clear cytotoxic lipid peroxides, these cytotoxic products may damage the stability of lipid bilayer, leading to oxidation, damage, and leakage of the lipid membranes, as well as the formation of pores on the cell membrane^[95]^, ultimately resulting in cell death. Therefore, ferroptosis plays a central role in the context of TGF-β1 in cancer, highlighting the significance of regulating lipid peroxidation and iron metabolism for intervening ferroptosis in cancer therapeutics.

## The correlation between ferroptosis and diseases

**Figure 3:**
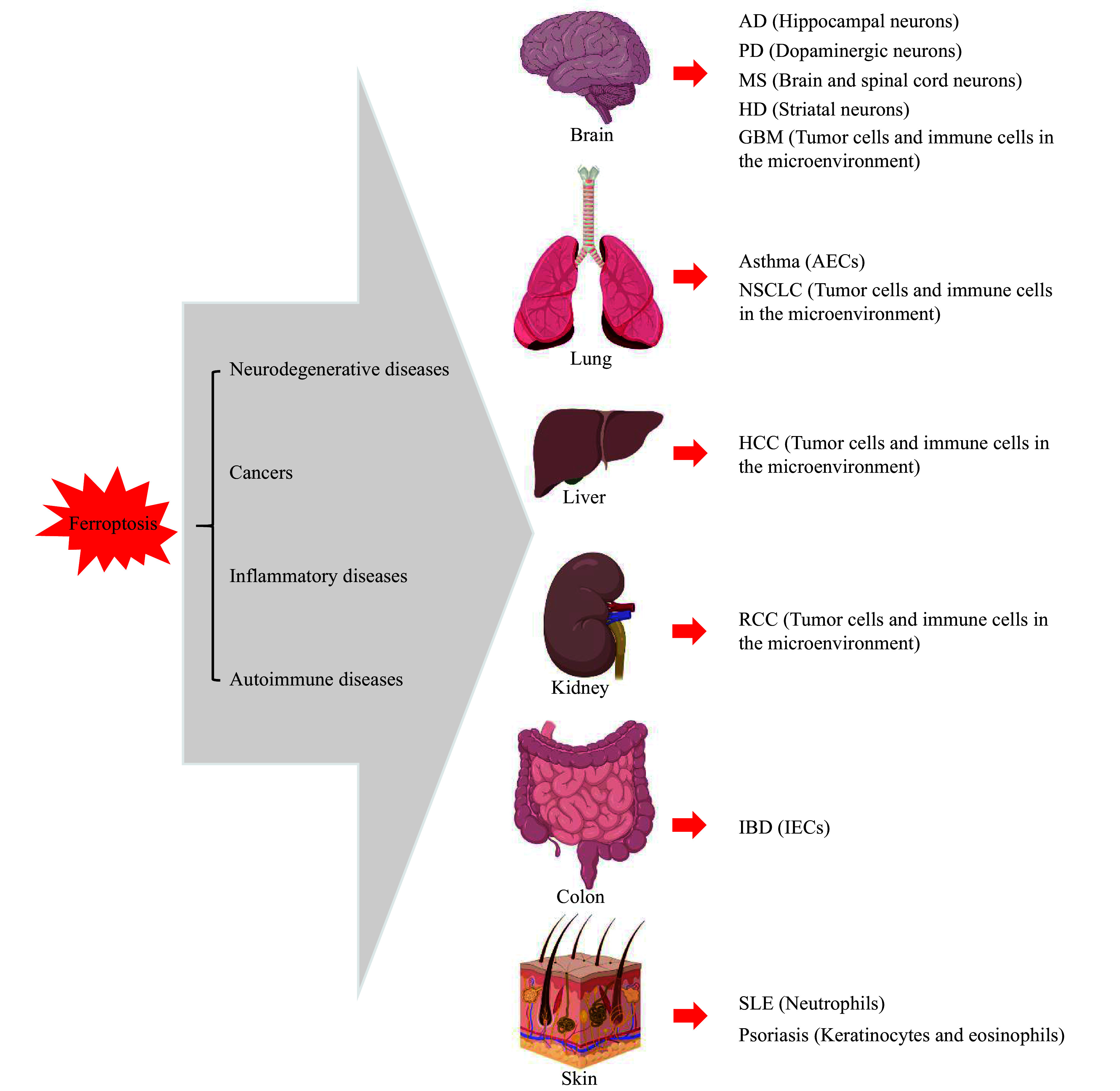
Associations of ferroptosis with chronic diseases.

In animal models, inhibiting ferroptosis may delay the progression of neurodegenerative diseases, autoimmune diseases, and inflammatory diseases (***Fig. 3***). On the other hand, inducing the ferroptosis of cancer cells holds a promise for the treatment of cancers that are unresponsive to the standard therapies.

### Ferroptosis and cancers

HCC is characterized by high malignancy, rapid progression, and poor prognosis. Therefore, inducing cytotoxicity in HCC tumor cells is an effective strategy for HCC treatment. Sorafenib is a multi-kinase inhibitor that may inhibit tumor cell proliferation and tumor angiogenesis, and promote apoptosis of various tumor cells^[96]^. Currently, oral administration of sorafenib has been used to treat HCC^[25]^. However, contrary to previous understanding of sorafenib-induced tumor cell apoptosis, Louandre *et al*^[25]^ demonstrated that the cell death induced by sorafenib alone was ferroptosis, and that this type of cell death was blocked by ferroptosis inhibitor deferasirox (DFX). Another study has also demonstrated that sorafenib functions similarly to erastin, a known ferroptosis inducer by blocking system X_c_^–^ and inhibiting GSH synthesis in HCC cells, thereby promoting ferroptosis in HCC^[26]^. NRF2 is likely another important regulator of antioxidant defense, because inhibition of the p62-KEAP1-NRF2 antioxidant signaling pathway may significantly enhance the cytotoxicity of sorafenib on HCC cells both *in vivo* and *in vitro*^[27]^.

Glioma, also known as glioblastoma, is the most common tumor type of the central nervous system and is treated with temozolomide, the most commonly used first-line drug for chemotherapy, but the therapeutic effect of temozolomide is limited^[97]^. Studies have shown that using the ferroptosis inducer erastin or directly silencing the expression of SLC7A11 protein in system X_c_^–^ induces ferroptosis in glioma cells, and increases the sensitivity of glioma cells to temozolomide, thereby promoting the killing effect of temozolomide on tumor cells^[98–99]^. Activating transcription factor 4 (ATF4) may also be a potential therapeutic target for glioma, because the expression and activation of ATF4 are enhanced in glioma cells, leading to a significant up-regulation of system X_c_^–^ expression, thereby reducing the sensitivity of tumor cells to ferroptosis, and thus promoting tumor cell proliferation, tumor angiogenesis, and other malignant characteristics^[28]^. In contrast, ATF4 knockdown may induce ferroptosis and control tumor parenchyma and blood vessel growth^[28]^.

Additional studies have shown that the expression level of GPX4 is significantly higher in tumor tissues than in normal tissues and positively correlated with the proliferation, invasion, and metastasis of the tumor^[100–101]^.

Clear cell renal cell carcinoma (ccRCC) is the most common type of kidney cancer, and its clinical treatment still faces significant challenges. One study has demonstrated an association between Kruppel-like factor 2 (KLF2) and GPX4 in ccRCC^[29]^. KLF2 functions as a transcriptional repressor and promotes ferroptosis by downregulating the GPX4 expression, thus inhibiting cell migration, invasion, and metastasis of ccRCC both *in vitro* and *in vivo*^[29]^. Downregulation of KLF2 has been shown to significantly inhibit ferroptosis and promote tumor cell invasion by weakening transcriptional repression of GPX4^[29]^. In addition, SLC7A11 is significantly upregulated in all three subtypes of kidney cancer, including chromophobe renal cell carcinoma, ccRCC, and papillary renal cell carcinoma^[102]^. Xu *et al*^[103]^ found that overexpression of SLC7A11 led to a reduced immune cell infiltration in tumor tissues, an impaired antigen-presenting cell function, and suppressed anti-tumor immune responses. At the same time, it was observed that the GPX4 expression was significantly increased, hindering ferroptosis and promoting renal cancer cell proliferation, migration, and invasion^[103]^.

Non-small cell lung carcinoma (NSCLC) is the main subtype of lung cancer, and long non-coding RNA (lncRNA) is involved in the development of NSCLC by regulating genes related to ferroptosis. For example, the tankyrase inhibitor XAV939 targets the lncRNA-regulated gene, downregulates the SLC7A11 expression, disrupts the redox homeostasis within cells, induces ferroptosis, and inhibits the development of NSCLC^[104]^. Nuclear enriched transcript 1 is an oncogenic lncRNA that targets and suppresses the expression of *ACSL4* mRNA, thereby reducing the sensitivity of NSCLC cells to ferroptosis and promoting tumor cell proliferation and metastasis^[105]^.

Cancer cells generally have a lower degree of unsaturated fatty acids, because a higher degree of unsaturated fatty acids results in a greater susceptibility of the cell membrane to lipid peroxidation and subsequent cell death^[106]^. One study has demonstrated a significant increase in long-chain polyunsaturated lipids in reactive tumors after the anti-programmed cell death protein-1 (PD-1) therapy^[107]^. Moreover, the combined treatment of low-dose AA and anti-PD-L1 therapy reduced tumor growth more potently, compared with either treatment alone^[108]^, implying that the tumor-killing effect of anti-PD-1/PD-L1 therapy may be correlated with ferroptosis. Regulating methionine intake also affects tumor cell ferroptosis, and a sustained dietary methionine deficiency inhibits tumor ferroptosis, while an intermittent deficiency promotes tumor ferroptosis and enhances the sensitivity of tumor cells to CD8^+^ T cells^[109]^. The triple combination therapy of intermittent methionine deficiency in the diet, ferroptosis inducers, and PD-1 blockers shows superior anti-tumor effects^[109]^. In conclusion, the combination of anti-PD-1/PD-L1 therapy with ferroptosis inducers may effectively exert an anti-tumor effect. However, it is still under investigation whether the immune checkpoint therapy exerts anti-tumor effects through the mechanism of ferroptosis and how it participates in the regulation of ferroptosis in tumor cells. Future research is needed in this area.

### Ferroptosis and neurodegenerative diseases

The disruption of iron homeostasis and the dysregulation of cellular redox state play crucial roles in the initiation and progression of neurodegenerative diseases, including AD, PD, Huntington's disease (HD), and multiple sclerosis (MS).

#### Ferroptosis and AD

AD is a primary irreversible degenerative brain disease, and there are many hypotheses about its pathogenesis, of which the amyloid beta-protein (Aβ) cascade hypothesis has been widely recognized^[110]^. Aβ is the degradation product of amyloid precursor protein, and brain regions that are rich in Aβ show significantly increased levels of lipid peroxide, such as 4-HNE, implying that Aβ deposition may be related to lipid peroxidation^[111]^. The iron homeostasis imbalance has also been found in AD, with the *in vivo* magnetic resonance imaging showing a significant increase in iron content in the hippocampus of AD patients^[30]^. Ferroportin is the only critical channel protein for iron efflux in cells^[31]^. It has been demonstrated that the expression of ferroportin is significantly down-regulated in the hippocampus and frontal cortex of the brain in both AD patients and mouse models, which causes intracellular iron deposition, inducing neuronal ferroptosis and resulting in memory impairment in patients^[31]^. In addition, Galante *et al*^[112]^ also demonstrated iron's capacity to accelerate the aggregation of Aβ, promote the formation of amyloid plaques, and increase their cellular toxicity by *in vitro* experiments.

#### Ferroptosis and PD

PD is a common neurodegenerative disease in middle-aged and elderly individuals. The main pathological mechanism involves the degeneration and death of dopaminergic neurons in the compact region of the substantia nigra that regulates motor functions^[113]^. In addition to the regulated cell death, such as necroptosis and pyroptosis, which are involved in PD progression^[114–115]^, the ferroptosis-related markers have also been discovered in PD. Abnormal iron accumulation was observed in the compact region of the substantia nigra in the early stages of PD, and as the disease progresses, iron accumulation gradually appeared in other regions of the brain, such as the putamen, caudate nucleus, and globus pallidus^[116]^. Additionally, iron concentration is positively correlated with the severity of PD^[117]^. Currently, it has been shown that oral administration of DFP effectively reduces the accumulated iron levels in the substantia nigra by chelating free Fe^2+[118]^. This approach may also reduce the damage of oxidative stress to dopamine nerve cells, increase dopamine levels in the striatum, and improve motor function, and such a therapeutic effect may be sustained for a relatively long duration^[118]^. Furthermore, a reduction in the PUFA content and a substantial elevation in malondialdehyde and 4-hydroxynonenal content, both of which are intermediate molecules in lipid peroxidation with cytotoxic effects, have been identified in the substantia nigra of PD patients, implying that severe lipid peroxidation reactions may occur in dopamine nerve cells located in the substantia nigra^[32]^. It has been also shown that regulating SLC7A11 to enhance the cystine transport activity of the system X_c_^–^ promotes the synthesis of GSH, thereby enhancing the clearance of lipid peroxidation products and delaying the neurodegeneration of brain tissue^[73]^. The increasing comprehension of the mechanisms underlying ferroptosis in PD offers some hope for the development of effective therapies to halt or even reverse neurodegeneration.

#### Ferroptosis and MS

MS is an autoimmune disease characterized by multiple scattered, plaque-like lesions of inflammatory demyelination in the white matter of the central nervous system. There have been multiple research findings indicating that ferroptosis is involved in the pathogenesis of MS. In the neurons of the brain and spinal cord of both MS patients and the experimental autoimmune encephalomyelitis (EAE) mouse model, the expression levels of GPX4 are significantly decreased, GSH is depleted, PUFA content is reduced, and the contents of lipid peroxidation products (*i.e.*, malondialdehyde and 4-hydroxynonenal) are significantly increased as well^[119]^. Iron has also been found to contribute to the pathogenesis and progression of MS because of its accumulation in the human brain^[120]^. Studies found that ferroptotic neurons promoted the activation of T cells, thus exacerbating the symptoms of EAE. For example, in EAE mouse models, Luoqian *et al*^[121]^ demonstrated that ferroptosis occurred during both early and late stages of the disease, and that in neurons undergoing ferroptosis, Th1 and Th17 cells were selectively activated through modulation of the TCR signaling pathway, exacerbating the progression of EAE. Liproxstatin-1 (Lip-1) is a potent inhibitor of ferroptosis, and the treatment with Lip-1 in EAE results in decreased clinical scores, symptom alleviation, and reduced production of IFN-γ and IL-17 by T cells isolated from the central nervous system, indicating the impaired activation of Th1 and Th17 cells^[121]^. However, future research is needed to determine which substances are released by ferroptotic neurons to selectively activate T cells.

#### Ferroptosis and HD

HD is a rare autosomal dominant inherited disease, clinically characterized by chorea, progressive dementia, and emotional and psychiatric disorders. A variety of ferroptosis markers are observed in HD, such as a decrease in both GSH content and GPX4 activity^[122]^. At present, there is no specific treatment for HD, nor is there any effective medicine to slow the progression of the disease, except for symptomatic treatments. The role of ferroptosis inhibitors in HD has been demonstrated in animal models. For example, treating the striatum of rats with HD using the ferroptosis inhibitor ferrostatin-1 (Fer-1) effectively reduced lipid peroxidation levels, prevented neuronal death, and thereby slowed down the progression of the disease^[123]^. Future clinical trials are anticipated to evaluate the therapeutic efficacy of these inhibitors and explore the potential for effective therapies that may alleviate the course of HD and enhance patients' well-being.

### Ferroptosis and inflammatory diseases

It is known that ferroptotic cells may activate the innate immune system by releasing inflammation-related damage-associated molecular patterns, thereby triggering an inflammatory response. A moderate inflammatory response is conducive to the maintenance of body homeostasis, while an excessive inflammatory response causes inflammatory diseases and damage to the body. Although the pathological mechanisms of inflammatory diseases are complex, with the in-depth study of ferroptosis in recent years, ferroptosis is shown to be involved in the pathogenesis of various inflammatory diseases.

#### Ferroptosis and inflammatory bowel disease

Inflammatory bowel disease is a group of chronic, non-specific, inflammatory gastrointestinal diseases, mainly including Crohn's disease and ulcerative colitis. The occurrence of such diseases is correlated with the imbalance of intestinal homeostasis^[124–127]^. Studies have shown that ferroptosis is involved in the pathogenesis of Crohn's disease and ulcerative colitis. For example, Xu *et al*^[33]^ observed an increase in Fe^2+^ and ROS levels as well as mitochondrial atrophy in intestinal epithelial cells in human ulcerative colitis tissues as well as in the dextran sulfate sodium-induced colitis mouse model, and this effect was suppressed by the ferroptosis inhibitor Fer-1. In the mouse Crohn's disease model induced by TNBS, ferroptosis characteristics were also observed^[34]^. These findings imply that targeting the ferroptosis pathways may lead to innovative therapeutic strategies for inflammatory bowel disease, particularly for effective disease management and better outcomes of the patients.

#### Ferroptosis and asthma

Asthma is a chronic inflammatory airway disease characterized primarily by a Th2-type inflammatory response. The RNA-seq analysis of airway smooth muscle cells in asthmatic patients revealed an overexpression of ferroptosis-related markers, indicating a correlation between ferroptosis and asthma^[128]^. In one study of the IL-4/IL-13-driven Th2-type inflammatory responses, human airway epithelial cells were found to significantly upregulate 15-LOX expression that binds with PEBP1 to promote the generation of AA/AdA-PE-OOH, inducing ferroptosis of airway epithelial cells^[129]^. The upregulation of 15-LOX expression was also detected in airway epithelial cells of both stable and severe asthma patients, and the expression level was positively correlated with the severity of asthma^[129]^. In addition, treatment with ferroptosis inducers in a Th2-type airway inflammation animal model induced eosinophil death and effectively alleviated eosinophilic airway inflammation^[130]^. These results imply that ferroptosis of airway epithelial cells is involved in the inflammatory response of asthma and plays an important role in the pathological progression of asthma.

### Ferroptosis and autoimmune diseases

#### Ferroptosis and psoriasis

Psoriasis is a common chronic autoimmune inflammatory skin disease, characterized by well-defined red patches of various sizes and shapes on the affected skin, covered with multiple layers of silver-white scales. In the skin lesions of psoriasis patients, the expression of ferritin light chain and ferritin heavy chain 1 in keratinocytes was found to be downregulated, while the expression of TFR increased^[35]^. These indicate that the iron content entering the keratinocytes is elevated, while the iron storage capacity is reduced, leading to a high level of Fe^2+^ ion within the cells and resulting in ferroptosis. Shou *et al*^[35]^ also demonstrated that keratinocytes were highly sensitive to ferroptosis in a mouse model of psoriasis induced by imiquimod. When treated with a ferroptosis inhibitor Fer-1, ferroptosis in keratinocytes was inhibited, and the levels of proinflammatory cytokines TNF-α, IL-6, IL-1α, IL-1β, IL-17, IL-22, and IL-23 were reduced, and the inflammation of psoriasis-like skin was alleviated^[35]^. These observations underscore the significance of targeting the ferroptosis pathway in exploring novel therapeutic avenues for psoriasis management.

#### Ferroptosis and systemic lupus erythematosus

Systemic lupus erythematosus is an autoimmune disease characterized by pathological involvement of multiple organs, including the skin, serous membranes, joints, and kidneys. Previous research demonstrated that in patients with active systemic lupus erythematosus, serum autoreactive IgG and type Ⅰ interferon activated the neutrophil calcium/calmodulin kinase Ⅳ/cAMP response element modulator pathway, leading to transcriptional repression of GPX4 and resulting in intracellular lipid peroxidation and spontaneous ferroptosis in neutrophils^[36]^. Moreover, in a lupus-prone mouse model, treatment with the ferroptosis inhibitor Lip-1 and iron chelator deferoxamine (DFO) was shown to effectively inhibit neutrophil ferroptosis and significantly alleviate disease progression^[36]^. These findings imply that neutrophil ferroptosis mechanisms are involved in the pathogenesis of systemic lupus erythematosus.

## Drugs that target ferroptosis

Drugs targeting ferroptosis are a focal point of current pharmacological research. Categorized broadly as inducers or inhibitors, these agents are designed to either trigger or suppress the lipid peroxidation cascade, respectively. Ferroptosis inducers hold some promise in oncologic treatments by selectively inducing cell death in cancer cells, while inhibitors offer neuroprotective and anti-ischemic strategies by mitigating uncontrolled cellular peroxidation. This nuanced modulation of ferroptosis represents a promising therapeutic approach across diverse diseases. Here, we present a comprehensive overview through a detailed table (***Table 2***), highlighting the applications of ferroptosis-targeting drugs in animal disease models and the advancements in clinical trials, offering a more intuitive understanding of their use in diverse disease models and recent clinical trial progress.

**Table 2:** Ferroptosis-targeting drugs: applications in animal disease models and advancements in clinical trials

Drugs	Mechanism	Animal disease models	Clinical indication	Clinical trial phase	NCT#	Status
Ferroptosis inducers						
(1S,3R) -RSL3	Blocking GPX4 activity	DLBCLs^[134]^, RCC^[134]^, CRC^[135]^	N/A	N/A	N/A	N/A
FIN56	Degrading GPX4 Consuming CoQ_10_	GBM^[137]^	N/A	N/A	N/A	N/A
Erastin	Inhibiting system X_c_^–^ activity Blocking cystine uptake Interfering with GSH synthesis	NSCLC^[140]^, GBM^[98, 141]^	N/A	N/A	N/A	N/A
Sulfasalazine	Inhibiting system X_c_^–^ activity Blocking cystine uptake Interfering with GSH synthesis	Pancreatic cancer^[142]^	Metastatic colorectal cancer	Ⅲ	NCT06134388	Recruiting
Sorafenib	Inhibiting system X_c_^–^ activity Blocking cystine uptake Interfering with GSH synthesis	HCC^[25]^	NSCLC	Ⅱ	NCT00098254	Completed
Curcumin	Blocking cystine uptake Interfering with GSH synthesis	NSCLC^[143]^	Cervical cancer	N/A	NCT06080841	Recruiting
ZVINPs	Triggering iron overload Triggering the Fenton reaction	OSCC^[132]^	N/A	N/A	N/A	N/A
Fe_3_O_4_ porous nanoparticle carrier	Triggering iron overload Triggering Fenton reaction Carrying the chemotherapy drug	HCC^[133]^	N/A	N/A	N/A	N/A
Acetaminophen	Inhibiting system X_c_^–^ activity Depleting GSH	NSCLC^[144]^	Acute respiratory distress syndrome	Ⅱ	NCT04291508	Recruiting
Ferroptosis inhibitors						
Fer-1	Combining with LOOH Inhibiting lipid peroxidation	HD^[123]^, IBD^[33]^	N/A	N/A	N/A	N/A
Lip-1	Combining with LOOH Inhibiting lipid peroxidation	EAE^[121]^	N/A	N/A	N/A	N/A
Baicalein	12/15-Lox inhibitor Inhibiting lipid peroxidation	AD^[150]^, PTE^[151]^, Osteoarthritis^[152]^	Influenza	Ⅱ	NCT03830684	Unknown
DFO	Chelating iron ions Inhibiting the Fenton reaction	TSCI^[160]^	Ischemic stroke	Ⅱ	NCT00777140	Completed
DFX	Chelating iron ions Inhibiting the Fenton reaction	IBD^[159]^	Myelodysplasia	Ⅱ	NCT03387475	Recruiting
DFP	Chelating iron ions Inhibiting the Fenton reaction	PD^[118]^	Aneurysmal subarachnoid hemorrhage	Ⅱ	NCT04566991	Recruiting
Edaravone	Free radical scavenger Inhibiting lipid peroxidation	Chronic social defeat stress^[154]^	Acute ischemic stroke	Ⅲ	NCT02430350	Completed
Rosiglitazone	ACSL4 inhibitor	Intestinal ischemia/reperfusion^[156]^	Inflammatory bowel disease	Ⅱ	NCT00065065	Completed
N-acetylcysteine	Promoting GSH synthesis	Diabetic nephropathy^[157]^	Diabetic neuropathies	Ⅳ	NCT04766450	Recruiting
Abbreviations: ACSL4, acyl-CoA synthetase long-chain family member 4; AD, Alzheimer's disease; CoQ (ubiquinone), oxidised form coenzyme Q_10_; CRC, colorectal cancer; DLBCLs, diffuse large B cell lymphomas; DFO, deferoxamine; DFX, deferasirox; DFP, deferiprone; EAE, experimental autoimmune encephalomyelitis; Fer-1, ferrostatin-1; GBM, glioblastoma; GPX4, glutathione peroxidase 4; GSH, glutathione; HCC, hepatocellular carcinoma; HD, Huntington's disease; IBD, inflammatory bowel disease; 12/15-LOX, lipoxygenase 12/15; LOOH, lipid hydroperoxides; Lip-1, Liproxstatin-1; N/A, non-applicable; NCT, National Clinical Trials; NSCLC, non-small cell lung carcinoma; ZVINPs, zero-valent iron nanoparticles; OSCC, oral squamous cell carcinoma; PD, Parkinson's disease; PTE, posttraumatic epilepsy; RCC, renal cell carcinomas; TSCI, traumatic spinal cord injury.						

### Ferroptosis inducers

Ferroptosis inducers primarily target cancer cells, with GPX4 inhibitors and system X_c_^–^ inhibitors being the main classes^[131]^. Nanotechnology offers exciting possibilities for drug delivery, monitoring, and modulating iron metabolism, thereby enhancing the efficacy and specificity of ferroptosis-based anti-tumor therapy^[132–133]^. This synergistic integration of ferroptosis inducers with nanotechnology is poised to reshape the landscape of cancer treatment, paving the way for more effective and personalized therapeutic interventions.

#### GPX4 inhibitors

The (1S,3R)-RSL3 is an inducer of ferroptosis and acts as an inhibitor of GPX4. It covalently binds to the selenocysteine residue 46 within the active site of GPX4 through its electrophilic chloroacetamide structure by alkylating it, leading to irreversible inactivation of GPX4^[134]^. Studies showed that RSL3 effectively induced tumor cell ferroptosis in various tumor models, such as diffuse large B cell lymphomas, renal cell carcinomas, and colorectal cancer^[134–135]^. In addition, Shimada *et al*^[136]^ identified and named another ferroptosis inducer, FIN56. This compound induces GPX4 degradation, binds to and activates squalene synthase, and depletes CoQ_10_ through the squalene synthase-mevalonate pathway, thereby resulting in the decreased function of the GSH-GPX4 antioxidant system and CoQ_10_-dependent antioxidant system, and ultimately inducing ferroptosis^[136]^. Zhang *et al*^[137]^ also demonstrated the significant cytotoxic effect of FIN56 on glioblastoma using a nude mouse tumor model.

#### System X_c_^–^ inhibitors

Erastin induces tumor cell ferroptosis by directly inhibiting the activity of system X_c_^–[138]^. The low-dose and short-term erastin treatments have been shown to efficiently induce ferroptosis in various types of tumor cells, with a rapid and long-lasting effect^[139]^. Furthermore, erastin was also used in combination with other chemotherapeutic agents to increase the sensitivity of cancer cells to chemotherapy. Studies have demonstrated that the combination of erastin with cisplatin and temozolomide reduces the cytotoxic effects of these chemotherapy drugs on NSCLC and glioblastoma in animal tumor models^[98,140–141]^. Given the excellent effect of erastin-induced ferroptosis in animal models, it is expected that it will be useful in the treatment of cancer.

Other drugs with similar mechanisms include sulfasalazine, sorafenib, curcumin, and acetaminophen, whose application effects have been demonstrated in animal tumor models^[25,142–144]^, with sulfasalazine, as a broad-spectrum antibacterial agent; sorafenib, as a traditional chemotherapy drug; and curcumin as an anti-inflammatory drug. These drugs have all been used in different fields of clinical practice, but their clinical effects on tumor progression through modulation of ferroptosis have not been evaluated yet.

#### Nanomaterials

Nanomaterials possess unique advantages in drug delivery. Using nanomaterials to deliver iron to the tumor microenvironment may effectively promote ferroptosis of tumor cells by inducing the Fenton reaction. At present, zero-valent iron nanoparticles have been demonstrated to induce ferroptosis in human oral squamous cell carcinoma cells *in vitro*, thereby facilitating the clearance of tumor cells^[132]^. Iron-based nanomaterials may also carry chemotherapeutic drugs to improve the therapeutic effect of traditional chemotherapy and achieve synergistic treatment. For example, Xu *et al*^[133]^ synthesized a mangan-doped Fe_3_O_4_ porous nanoparticle carrier capable of delivering the chemotherapy drug Adriamycin. Upon reaching the tumor site, this carrier releases iron ions and Adriamycin, thereby promoting the killing of tumor cells by inducing the Fenton reaction and interfering with the nucleic acid synthesis of tumor cells^[133]^. In addition to the iron-based nanomaterials mentioned above, non-iron-based nanomaterials may also be used to induce ferroptosis in tumors, primarily through mechanisms such as depleting intracellular GSH and delivering ferroptosis inducers. At present, the application effects of the aforementioned nanomaterials have only been demonstrated in animal experiments and *in vitro* studies. Future research is still needed to determine their potential clinical applications.

Apart from the ferroptosis inducers mentioned above, targeting the inhibition of FSP1 activity may also induce ferroptosis in tumor cells. For example, the 3-phenylquinoline ketone compounds (icFSP1), as inhibitors of FSP1, exhibit distinct characteristics, compared with the existing FSP1 inhibitors^[145]^. Instead of competitively inhibiting the enzyme activity, icFSP1 promotes ferroptosis by triggering the dissociation of FSP1 from the membrane and forming phase separation-associated aggregates^[145]^.

### Ferroptosis inhibitors

The existing ferroptosis inhibitors primarily function through multiple mechanisms, including scavenging of free radicals, interruption of lipid peroxidation, and modulation of free iron content, thereby exerting a protective effect on cells. These inhibitors effectively neutralize intracellular free radicals to prevent them from detrimental damage to cell membranes and organelles, thus safeguarding cells against the onslaught of ferroptosis. Additionally, these inhibitors hinder the chain reaction of lipid peroxidation, mitigating oxidative injury to cell membranes and preserving cellular integrity and stability. Furthermore, by lowering the levels of intracellular free iron, these inhibitors diminish the catalytic oxidation of cellular components by iron ions, thereby mitigating the risk of ferroptosis. The synergistic interplay among these mechanisms underscores the significant value of current ferroptosis inhibitors in countering oxidative stress and ferroptosis while ensuring cellular survival and functionality.

#### Inhibitors of lipid peroxidation

Fer-1, as a lipid peroxidation scavenger, exerts its action dependent on the cycloheximide structure, and it directly inhibits lipid peroxidation reactions by selectively binding to LOOH^[123]^. Fer-1 may also induce the upregulation of GPX4 and NRF2 expression, thereby enhancing cellular resistance to oxidative stress and inhibiting ferroptosis^[146]^. Currently, the therapeutic efficacy of Fer-1 has been demonstrated in animal models of HD and inflammatory bowel disease^[33,123]^. Other compounds with similar effects include Lip-1, which was initially discovered through high-throughput screening^[16]^. Subsequent molecular structure-activity studies demonstrated that the aromatic amine structure was essential for the antioxidant activity of these ferroptosis inhibitors^[147–148]^. Thus, Fer-1 and Lip-1 are called arylamine antioxidants because of their mechanisms of action.

Baicalein is a flavonoid compound that exhibits antiplatelet aggregation properties and improves cerebral blood circulation, and is used to treat paralysis following cerebrovascular diseases. One *in vitro* experiment showed that baicalein acted as a selective 12/15-Lox inhibitor^[149]^, inhibiting the enzymatic synthesis pathway of LOOH and hindering lipid peroxidation. This drug exhibits significant potential in improving the cognitive function of AD in mice models, reducing Aβ deposition, and inhibiting tau protein phosphorylation^[150]^. The neuroprotective effect of baicalein on posttraumatic epilepsy by inhibiting ferroptosis has been demonstrated in the mouse model of iron chloride-induced posttraumatic epilepsy^[151]^. Animal studies also demonstrated that baicalein limited the development of osteoarthritis by inhibiting ferroptosis in chondrocytes^[152–153]^. However, the therapeutic effect of baicalein targeting the ferroptosis mechanism on related diseases has not been clinically evaluated.

Edaravone, a clinically approved free radical scavenger for the treatment of acute ischemic stroke and amyotrophic lateral sclerosis, exhibits resistance to ferroptosis^[154]^. Moreover, 2-methyl-5H-benzo[d]pyrazolo[5,1-b][1,3]oxazin-5-imine, a novel small-molecule compound structurally similar to edaravone, has a stronger inhibitory effect on oxidative stress^[155]^. Additionally, rosiglitazone, which is clinically used to treat type 2 diabetes, has also been shown to block ferroptosis by selectively inhibiting ACSL4^[156]^. N-acetylcysteine also exerts its inhibitory effects on lipid peroxidation through the promotion of GSH synthesis^[157]^. These aforementioned studies indicate a compelling scientific hypothesis that certain widely used clinical medications may possess the capability of therapeutically intervening in disease processes by precisely targeting the cellular mechanism of ferroptosis. This notion is supported by accumulating evidence indicating that modulation of ferroptosis-related pathways may potentially offer novel therapeutic strategies for a broad range of pathological conditions.

#### Iron chelators

Common iron chelators in clinical practice include DFP, DFO, and DFX, which bind to iron in a ratio of 3∶1, 1∶1, and 2∶1, respectively^[158]^. At present, clinical application of iron chelators is mainly to treat iron overload diseases, such as β-thalassemia and sickle cell anemia^[158]^. Several animal experiments have shown that iron chelators inhibited ferroptosis by reducing iron concentration. For example, in a dextran sulfate sodium-induced ulcerative colitis mouse model, DFX treatment effectively inhibited ferroptosis, ameliorated intestinal inflammation, and increased the abundance of beneficial short-chain fatty acids-producing bacteria^[159]^. In addition, the therapeutic efficacy of DFP and DFO has been demonstrated in animal models of PD^[118]^ and traumatic spinal cord injury^[160]^.

Additionally, Yang *et al*^[161]^ identified a non-classical small molecule ferroptosis inhibitor, YL-939, that is neither an antioxidant nor an iron chelator through compound screening experiments. Mechanistically, YL-939, by binding to its biological target prohibitin 2, promotes the expression of ferritin, hence reducing the iron content and inhibiting ferroptosis^[161]^.

## Conclusions and perspectives

Since 2012, when the concept of ferroptosis was put forward, the mechanisms underlying ferroptosis and its resistance have been extensively investigated. Ferroptosis-related metabolites, protein molecules, and organelles together form a highly flexible regulatory network to participate in the regulation of ferroptosis. Furthermore, ferroptosis is associated with many diseases, including cancer, neurodegeneration, inflammation, and autoimmunity. Iron metabolism disorders, GSH and GPX4 depletion, and lipid peroxidation are the common characteristics of ferroptosis and inflammatory diseases. Based on these insights, the development of potent ferroptosis inhibitors is promising in the treatment of inflammatory diseases.

At present, ferroptosis has been a hot topic in the field of cancer, and designing drugs to target ferroptosis is also a promising strategy for cancer treatment. However, the research on ferroptosis involved in disease regulation is still in its infancy. Apart from the known signal pathways and regulatory mechanisms, are there other factors regulating ferroptosis? Are there more connections between ferroptosis regulatory signals and immune signaling pathways? Increasing evidence has shown the crosstalk between ferroptosis and other types of cell death; however, the specific markers of ferroptosis have not been fully defined, and the specific connection of ferroptosis to other types of cell death remains unknown. Whether ferroptosis occurs as an active process or as a passive process in response to metabolic disturbances or accompanied by other types of cell death remains to be further investigated. Furthermore, although numerous ferroptosis inducers and inhibitors have been identified and their therapeutic effects have been demonstrated in related disease models, the targets and potential side effects are still unknown, and their safety and effectiveness in clinical application remain to be further investigated.
